# The South East Texas Geriatric Workforce Enhancement Program: Reach, Teach, Innovate

**DOI:** 10.1093/geroni/igab046.110

**Published:** 2021-12-17

**Authors:** Ali Asghar-Ali

**Affiliations:** Baylor College of Medicine, Michael E. DeBakey VA Medical Center/Houston, Texas, United States

## Abstract

Through collaboration between academic and community partners, the South East Texas Geriatric Workforce Enhancement Program (SETx GWEP) aims to promulgate the 4Ms framework via a range of educational initiatives. The faculty and audience is interprofessional and diverse, representing the residents of South East Texas. Specific initiatives focus on Alzheimer’s disease and related dementias, elder abuse, geriatric mental health, patient priorities, transitions of care, and geriatric dental care. Training modalities include online modules, Project ECHO sessions, webinars, discussion forums, and simulation. During the COVID19 pandemic the SETx GWEP adapted to meet the needs of its stakeholders, including increasing the number of online activities, hosting town hall meetings, and developing training to address the impact of COVID19 on the older adult population. The SETxGWEP trained over 1000 people in 2020. To address healthcare disparities among older adults, SETx GWEP developed training on the practice of cultural humility in older adult care.

